# Examining the relationship between metformin dose and cancer survival: A SEER-Medicare analysis

**DOI:** 10.1371/journal.pone.0275681

**Published:** 2022-10-19

**Authors:** Lisa Scarton, Ara Jo, Zhigang Xie, LaToya J. O’Neal, Juan M. Munoz Pena, Thomas J. George, Jiang Bian

**Affiliations:** 1 Department of Family, Community, and Health Systems Science, College of Nursing, University of Florida, Gainesville, Florida, United States of America; 2 Department of Health Services Research, Management and Policy, College of Public Health and Health Professions, University of Florida, Gainesville, Florida, United States of America; 3 Department of Public Health, University of North Florida, Jacksonville, Florida, United States of America; 4 Institute of Food and Agricultural Sciences, University of Florida, Gainesville, Florida, United States of America; 5 Division of Endocrinology, Diabetes and Metabolism, Department of Medicine, College of Medicine, University of Florida, Gainesville, Florida, United States of America; 6 Division of Hematology and Oncology, College of Medicine, University of Florida, Gainesville, Florida, United States of America; 7 Health Outcomes and Biomedical Informatics, College of Medicine, University of Florida, Gainesville, Florida, United States of America; Universitá Sapienza di Roma, ITALY

## Abstract

Cancer is a major health problem in the U.S and type 2 diabetes mellitus (T2DM) is known to increase the risk for the development of many cancers. Metformin, a first-line therapy for treating T2DM, is increasingly being used for its anticancer effects; however, the literature is limited on the effect of metformin dose on overall survival in patients with stage IV cancer. Overall survival was defined as the time interval from the date of diagnosis to the last known follow-up or death from any cause. Subjects who were alive on December 31, 2016 were censored. In this cohort study we examined the relationship between metformin dose and overall survival in persons with both T2DM and stage IV lung, breast, colorectal, prostate, or pancreas cancers. We used a retrospective study design with Cox proportional hazards regression analysis of the 2007–2016 of the Surveillance Epidemiology and End Results-Medicare (SEER) dataset. Of the 7,725 patients, 2,981(38.5%) had been prescribed metformin. Patients who used metformin had significantly better overall survival in both unadjusted (Unadjusted HR, 0.73; 95% CI, 0.69–0.76; p < 0.001) and adjusted models (adjusted HR, 0.77; 95% CI, 0.73–0.81; p < 0.001). The overall survival between patients who took metformin with average daily dose ≥ 1000mg or < 1000mg were not statistically significant (aHR, 1.00; 95% CI, 0.93–1.08; p = 0.90). Metformin use regardless of dose is associated with increased overall survival in older adults with stage IV cancer.

## Introduction

Cancer is a major health problem in the U.S. and is the second leading cause of death for Americans [1]. Type 2 diabetes mellitus (T2DM), another major health problem, is known to increase the risk for the development of many of the most common cancers (liver, pancreas, colorectal, breast) [2, 3]. Further, in some cancers, T2DM is associated with advanced stage cancer at diagnosis [4], higher rates of mortality [5], and worse overall survival [5]. However, metformin, a first-line therapy for treating T2DM, is an oral hypoglycemic agent used to reduce blood glucose levels and also for off-label uses such as treatment of cardiovascular disease, metabolic syndrome, and obesity [6–8]. Additionally, metformin has been associated with improved overall survival (OS) among patients with diabetes and cancers commonly associated with T2DM such as breast, lung, or colorectal cancer [9–12]; however, less is known regarding the effects of dosage of metformin and OS.

The literature is limited and has mixed findings on the effect of metformin dose on OS in patients with stage IV cancer. A previous study found that synergistic effects of a combination with chemotherapeutic drugs and metformin at a lower dose improved survival rate. Particularly, the use of metformin contributed to decreasing toxicity of chemotherapeutic drugs [13]. A systematic review examined OS in patients with diabetes and pancreatic cancer and found patients with early or locally advanced stage pancreatic cancer had increased OS with metformin use but not those with metastatic pancreatic cancer [14]. Similarly, a study examining older adults with pancreatic cancer found the use of metformin did not improve OS compared to those who did not use metformin [15]. In contrast, two studies examining adults 65 and older with diabetes and stage IV lung cancer or colorectal cancer who used metformin revealed statistically significant improved OS compared to non-metformin users, although metformin dose was not examined [16, 17]. With these inconsistent findings and limited knowledge on the role metformin dose plays in OS further research is needed. The purpose of this study was to examine the relationship between metformin dose and OS in persons with T2DM and stage IV lung, breast, colorectal, prostate, or pancreas cancers using a large, population-based Surveillance Epidemiology and End Results-Medicare (SEER) cancer registry.

## Methods

This was a cohort study that used longitudinal Medicare claims data. The study sample consisted of patients with an initial primary diagnosis of stage IV lung, breast, colorectal, prostate, or pancreas cancer during 2007 to 2016. Out of 225,056 patients identified, patients were excluded if cancer diagnosis was identified through autopsy or death certificate, or no pathological confirmation (n = 24,903). Patients who developed a second cancer within 60 months or before December 31, 2016 were also excluded (n = 8,791). To fully capture the medical information of those patients, we excluded patients with Health Maintenance Organization (HMO) or without continuous fee-for-services Medicare Part A or Part B coverage from 12 months before cancer diagnosis to the last available date of track in the dataset (n = 94,051). Patients were also excluded if they died within 90 days of the incidence of the cancer diagnosis (n = 39,577) or age of diagnosis was less than 66 (n = 6,225) to ensure a minimum of one-year Medicare claims to determine the comorbidity status prior to cancer diagnosis. Based on inclusion and exclusion criteria, a total of 51,509 patients with one of the five cancers were identified. Of those 51,509 patients, those who were newly diagnosed with T2DM, within 30 days of the cancer diagnosis, were included in the analysis (n = 7,725; See Table 1).

**Table 1:** Baseline characteristics of patients with cancer (e.g., lung, prostate, colorectal, pancreas and breast cancer) using Medicare claims data, 2007–2016 (n = 7725).

%		High Dose Metformin (n = 1409)	Low Dose Metformin (n = 1572)	No Metformin (n = 4744)	P-value
Cancer type	Breast	16.1	20.0	63.9	<0.001
	Colorectal	18.0	21.4	60.7	
	Lung	17.2	20.8	62.0	
	Pancreas	21.4	16.6	62.1	
	Prostate	21.7	23.0	55.4	
Age Group	66–69	25.5	20.7	53.9	<0.001
	70–74	20.9	21.5	57.6	
	75–79	17.2	20.3	62.5	
	> = 80	11.2	19.1	69.8	
Sex	Male	20.9	19.9	59.3	<0.001
	Female	15.7	20.8	63.5	
Race/Ethnicity	Non-Hispanic White	18.2	19.9	61.9	<0.001
	Non-Hispanic Black	13.1	17.4	69.5	
	Hispanic	25.1	23.2	51.7	
	Others	19.5	26.0	54.6	
Marital Status	Single	17.7	19.8	62.6	<0.001
	Married	21.0	21.0	58.0	
	Other	14.7	19.8	65.5	
Poverty	= <25%	17.0	19.6	63.4	.17
	<25–50%	19.3	20.2	60.4	
	<50–75%	19.6	20.6	59.8	
	<75–100%	16.9	20.6	62.6	
Region	West	20.7	22.0	57.3	< .001
	Northeast	15.0	19.1	65.9	
	Midwest	17.0	19.3	63.8	
	South	18.3	19.7	62.0	
Comorbidity Score	1	22.2	25.2	52.7	<0.001
	2	21.9	21.4	56.8	
	3+	12.1	15.1	72.8	
Surgery	No	18.3	20.1	61.6	0.04
	Yes	17.1	26.7	56.2	
Radiation Therapy	No	17.7	19.5	62.8	0.001
	Yes	19.4	22.1	58.5	
Chemotherapy	No	13.1	17.3	69.6	<0.001
	Yes	21.2	22.1	56.7	

The primary outcome of OS was defined as the time interval from the date of diagnosis to the last known follow-up or death from any cause. Cancer death was determined using SEER data, which included primary cause of death abstracted from death certificates. Because of availability of the data based on the most recent SEER-Medicare linkage at the time of analyses, the last day of follow-up was December 31, 2016, for OS.

Medicare Part D data during 2007 to 2016 were used, including information on medications dispensed, drug potency, dispensing dates, and days of supply dispensed. Patients were defined as users of metformin on the day the medication was first dispensed after the cancer diagnosis and would remain in the user category throughout the follow-up.

Sociodemographic characteristics consisted of age at diagnosis, race, ethnicity, marital status, census region poverty, census region, Charlson Comorbidity score, surgery, radiation therapy, and chemotherapy. Age at the time of diagnosis was categorized into 4 groups (66–69, 70–74,75–79,80+). Race/ethnicity was categorized into four groups: Non-Hispanic White, Non-Hispanic Black, Hispanic, and other. Marital status was categorized into three groups: single, married, and other. Census region poverty refers to the percentage of residents living below the poverty level and was categorized into four groups (= <25%, <25–50%, <50–75%, <75–100%). Census region was categorized into West, Northeast, Midwest, and South. Klabunde modified Charlson Comorbidity score [18] was categorized into four groups (0, 1, 2, 3+). Surgery, radiation therapy, and chemotherapy were categorized into two groups (yes or no). All three treatments were provided within a year of stage IV cancer diagnosis.

### Metformin dosage

Metformin dosage was categorized into three levels, no metformin, lower dosage between 0 and 1000 mg/day (reference group) and higher dosage greater than 1000 mg/day. To test the effect of metformin, lower dosage which is the typical treatment for early diagnosis of patients with T2DM, was set as a reference group. The American Diabetes Association recommends a patient-centered approach to guide the dosing of glucose-lowering medication [19]. Metformin dosage is typically determined by the patient’s HbA1c or blood glucose levels, how well the patient tolerates metformin, and if the patient meets treatment goals [19]. We are using 1000mg/day as a threshold of daily use.

### Statistical analysis

We compared baseline patient characteristics by patients using metformin versus patients who were not using metformin using chi-square test (Table 1). Cox proportional-hazards models were performed to examine association of OS after a cancer diagnosis and metformin use. Unadjusted Cox proportional-hazards models for each selected predictor were performed and adjusted multivariable-adjusted Cox proportional-hazards models controlling for control variables (e.g., age, sex, race/ethnicity, marital status, poverty level, census region, Charlson Comorbidity score, surgery, radiation therapy, and chemotherapy) was conducted to test the association between metformin use and OS. Additionally, a metformin dosage-specific OS was performed using the same method. To explore differences in the relationship of metformin dosage and survival across race/ethnicity, subgroup analyses were performed. We conducted all analyses in SAS version 9.4 software (SAS Institute, Cary, NC). All statistical tests were 2-sided with statistical significance as p < 0.05.

## Results

A total of 7,725 patients with T2DM and at least one targeted cancer was included in the analysis. Of the 7,725 patients, 2,981(38.5%) had been prescribed metformin. Patients with prostate cancer (44.6%) were the largest group prescribed metformin compared to patients with other cancer types (Table 1). Patients aged 66–69 years, male, and Hispanics were more likely to be treated with metformin. Patients with higher comorbidity scores measured by Charlson index were significantly less likely to use metformin (p < .01). In terms of treatments, patients who undertook any form of treatment were less likely to be prescribed metformin. Hispanics were more likely to take metformin while Blacks were less likely compared to non-Hispanic Whites (Table 1). However, patients who had radiation therapy and chemotherapy showed significantly higher prescriptions of metformin compared to those without treatments (p < .01) whereas patients who had surgery did not show significant difference (p = .08).

In the multivariable Cox proportional hazard analyses for OS (Table 2), patients who used metformin had significantly better OS in both unadjusted (Unadjusted HR, 0.73; 95% CI, 0.69–0.76; p < 0.001) and adjusted models (adjusted HR, 0.77; 95% CI, 0.73–0.81; p < 0.001). In the adjusted model, undertaking surgery (aHR, 0.78; 95% CI, 0.68–0.89; p < 0.001), or receiving chemotherapy (aHR, 0.69; 95% CI, 0.65–0.72; p < 0.001) were associated with better OS. Receiving radiation had a negative association with survival.

**Table 2:** Multivariable-adjusted hazard ratios of overall survival between individuals taking metformin and those who didn’t take metformin.

		Unadjusted HR (95% CI)	Adjusted HR (95% CI)	P-value
Taking Metformin	No	1.00 (Reference group)	1.00 (Reference group)	
	Yes	0.73(0.69–0.76)	0.77(0.73–0.81)	< .001
Age Group	66–69	1.00 (Reference group)	1.00 (Reference group)	
	70–74	1.06(1.00–1.13)	1.08(1.01–1.15)	.021
	75–79	1.09(1.02–1.16)	1.06(0.99–1.14)	.073
	> = 80	1.15(1.07–1.22)	1.10(1.03–1.19)	.005
Sex	Male	1.00 (Reference group)	1.00 (Reference group)	
	Female	1.01(0.97–1.06)	0.86(0.82–0.91)	< .001
Race	Non-Hispanic White	1.00 (Reference group)	1.00 (Reference group)	
	Non-Hispanic Black	0.99(0.93–1.06)	0.96(0.90–1.03)	.30
	Hispanic	0.96(0.89–1.04)	0.99(0.91–1.08)	.79
	Others	0.91(0.84–0.99)	0.92(0.84–1.00)	.062
Marital Status	Single	1.00 (Reference group)	1.00 (Reference group)	
	Married	0.93(0.88–0.99)	0.92(0.87–0.98)	.007
	Other	0.99(0.93–1.05)	0.96(0.90–1.02)	.22
Poverty	= <25%	1.00 (Reference group)	1.00 (Reference group)	
	<25–50%	1.06(0.99–1.14)	1.07(1.00–1.15)	.051
	<50–75%	1.09(1.02–1.16)	1.06(0.99–1.14)	.099
	<75–100%	1.11(1.03–1.19)	1.07(0.99–1.15)	.099
Region	West	1.00 (Reference group)	1.00 (Reference group)	
	Northeast	0.98(0.92–1.04)	0.98(0.92–1.04)	.49
	Midwest	1.04(0.97–1.11)	1.02(0.94–1.09)	.69
	South	1.09(1.03–1.16)	1.07(1.00–114)	.37
Cancer	Breast	1.00 (Reference group)	1.00 (Reference group)	
	Colorectal	1.33(1.20–1.47)	1.32(1.18–1.48)	< .001
	Lung	1.92(1.76–2.11)	1.87(1.69–2.07)	< .001
	Prostate	0.81(0.72–0.91)	0.80(0.70–0.91)	.001
	Pancreas	2.58(2.33–2.86)	2.77(2.47–3.10)	< .001
Comorbidity Score	1	1.00 (Reference group)	1.00 (Reference group)	
	2	1.08(1.02–1.15)	1.03(0.97–1.09)	.39
	3+	1.21(1.15–1.27)	1.10(1.04–1.16)	.001
Surgery	No	1.00 (Reference group)	1.00 (Reference group)	
	Yes	0.59(0.52–0.66)	0.78(0.68–0.89)	< .001
Radiation Therapy	No	1.00 (Reference group)	1.00 (Reference group)	
	Yes	1.06(1.01–1.11)	1.08(1.03–1.14)	.003
Chemotherapy	No	1.00 (Reference group)	1.00 (Reference group)	
	Yes	0.69(0.65–0.72)	0.69(0.65–0.72)	< .001

**Fig 1:**
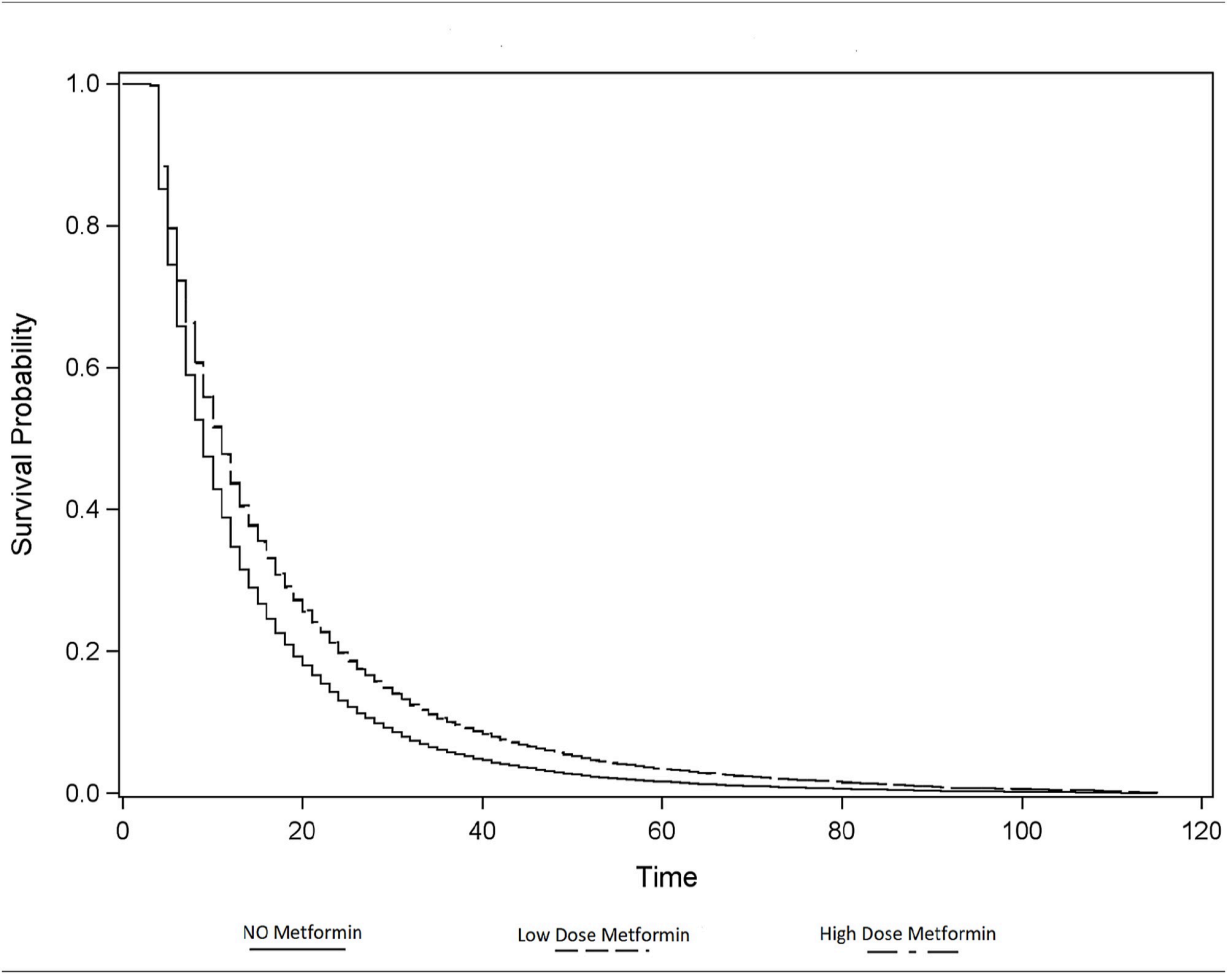
Kaplan-Meier curves of overall survival between patients with Metformin and those without Metformin.

Nevertheless, the OS between patients who took metformin with average daily dose over 1000mg and equal or less 1000mg were not statistically significant (aHR, 1.00; 95% CI, 0.93–1.08; p = 0.90). Fig 1 depicts Kaplan-Meier curves of OS between patients who had been prescribed metformin and those without metformin. Metformin use presented better OS regardless of dosage. Race/ethnicity did not play a role as a significant factor to predict better OS. Table 3 shows multivariable-adjusted hazard ratios of OS by subgroup of each cancer type between patients with metformin and those without metformin prescription. Patients who were prescribed metformin had significantly better OS in each subgroup of cancer. Patients with breast cancer who had metformin showed the most improvement in OS (aHR, 0.67; 95% CI, 0.56–0.82, p < .001). However, metformin dosage was not statistically associated with OS. Moreover, OS was not significantly different across race/ethnicity.

**Table 3:** Multivariable-adjusted hazard ratios of overall survival by Subgroup of each cancer type between patients with Metformin and those without Metformin.

	Adjusted HR (95% CI)	P-value
Breast Cancer		
Taking Metformin		
No	1.00	
Yes	0.67(0.56–0.82)	< .001
Colorectal Cancer		
Taking Metformin		
No	1.00	
Yes	0.78(0.69–0.88)	< .001
Lung Cancer		
Taking Metformin		
No	1.00	
Yes	0.77(0.72–0.82)	< .001
Pancreas Cancer		
Taking Metformin		
No	1.00	
Yes	0.82(0.72–0.93)	.002
Prostate Cancer		
Taking Metformin		
No	1.00	
Yes	0.74(0.62–0.88)	< .001

## Discussion

This population-based cohort study of patients with diabetes and stage IV cancers (including lung, breast, colorectal, prostate, or pancreas) adds to the limited knowledge on the relationship between metformin use, dose and OS. Our study found that patients with diabetes treated with surgery or receiving chemotherapy had better OS when also receiving metformin compared to those not receiving this agent. This is similar to the findings in a meta-analysis of patients with diabetes and pancreatic cancer where subgroup analysis revealed patients receiving surgery had increased OS; however, this was not consistent for those receiving chemotherapy or those with stage IV cancer [14]. Similarly, in another study with patients who have diabetes and stage IV lung cancer, no significant difference in OS was noted in patients receiving chemotherapy and metformin [16]. Other studies including RCTs has shown the benefit of the use of metformin in combination with chemotherapy in response to cancer treatment [20].

Findings from our study indicate that metformin use, regardless of dose, increases OS in older adults with these solid tumors. Previous literature has reported inconsistent results. Two meta-analyses for patients with stage I-IV breast and colorectal cancers found metformin use was associated with significantly prolonged OS [9, 10]. Improved OS can be explained by better biomarkers associated with metformin as well. A meta-analysis of RCTs for those with breast cancer showed significant improvement in insulin, glucose, and BMI despite no significant dose-response relationship between metformin and these biomarkers [21]. Moreover, a review paper speculated the anticancer protective effects of metformin “inhibited aerobic glycolysis in cells containing functional LKB1” [22]. Alternatively, a systematic review and meta-analysis examining patients with pancreatic cancer showed no significant relationship between metformin use and OS in patients with more advanced stage IV disease [14]. Metformin dose was not analyzed due to few studies reporting this information [9, 10, 14]. Together, it indicates that the impact of metformin may be limited or restricted to those patients being treated with curative intentions or with enough longevity to measure a clinically relevant benefit from the use of metformin. Importantly, as more evidence of anti-cancer benefit of metformin emerges, it will be important to identify an optimal dosage that is associated with improved cancer associated outcomes.

The protective effect of metformin may be associated with specific types of cancer and treatments. Our finding reported different effect size of metformin associated with specific types of cancer. As opposed, one meta-analysis study assessing the association between metformin and OS in patients with different cancer types and treatments reported mixed results [23]. Similar to our findings, the OS benefit of metformin use was present in prostate and colorectal cancer, but not in breast cancer. Moreover, its positive effect was explicit in patients with prostate cancer undergoing radical radiotherapy, consistent with the premise that early stage disease and/or longevity might be responsible for seeing the benefit. However, another study examining patients with locally advanced non-small cell lung cancer receiving both metformin and chemo/radiotherapy found no associations with improved OS [24]. It is still unclear what metformin doses and with which chemotherapeutic drugs might support an opportunity for synergistic benefits and why certain malignancies might be predisposed to preferentially benefit. Future studies should explore the relationship of types of chemotherapeutic drugs and metformin dosage in certain cancer types. Additionally, further research needs to be conducted to better understand dose response, including time-dependent cumulative response and the relationship to OS in persons with late stage cancers.

It is well established that metformin provides a better glucose response for T2DM and prediabetes in African Americans [25–27]. Despite this favorable association, our study failed to identify a significant difference in survival across race/ethnic groups. However, our finding is consistent with other published evidence failing to establish a positive association between metformin use and improved OS in African American cancer patients with diabetes [28]. Moreover, another study reported that metformin use is significantly and positively associated with aggressive prostate cancer particularly in African American patients with T2DM [29]. Those studies did not speculate the mechanism of mixed findings; however, there are some hypothetical reasons including genetic factors and medication adherence behavior in different race/ethnic groups [28]. It is difficult to conclude explicit benefits of metformin use in particular race/ethnic groups with these mixed findings. Future studies need to speculate physiological mechanism between metformin and cancer with respect to dose-response relationship.

### Limitations

Limitations of our study need to be considered. OS was used as a primary outcome as it was available in the claims data. The data did not provide treatment-specific survival. However, OS is the most objective and available endpoint to use. Future studies may use epidemiology study to investigate specific outcomes. In addition, as this study focused on the effect of metformin use on different types of cancer, it didn’t compare the effect of different T2DM treatment regimens on survival. Future studies need to scrutinize the comparative effectiveness of each T2DM regimens on overall survival. The dose of 1000mg daily was chosen as a threshold of metformin “use” since that is a fairly standard initial starting dose in clinical practice for T2DM patients with other medical problems (i.e., advanced cancer; [30]). Comparing metformin versus no metformin treatment for patients with advanced cancer is an oversimplified comparison that does not consider varying dosages or other aspects of diabetic complications or health, each of which are important factors that may impact OS. Second, confounding variables in any retrospective study may be present that can’t be fully accounted for or identified. We believe that the overall size of the study and utilization of an inclusive national database (SEER) helps to partially mitigate or diffuse some of these confounding biases, but they cannot be completely avoided. Third, the study was conducted using a time period of 2007 to 2016 which was deliberately chosen prior to the wide availability and FDA-approval of effective immunotherapies for several of the cancers studied, thereby limiting the impact of cancer therapeutic improvements over time on our survival analysis. Individual’s age 66 (n = 6,225) and younger were excluded at diagnosis of cancer to ensure a minimum of one-year Medicare claims to determine the comorbidity status prior to cancer diagnosis. It may not be generalizable to the entire cancer population and younger population. Finally, adherence is an important factor in controlling T2DM but was not able to be examined using this methodology. Future studies should evaluate the role of cancer patient diabetic treatment adherence on OS.

In conclusion, our study indicates metformin use is positively associated with improvements in OS in T2DM cancer patients; however, the benefit was not equal across diseases, stages of disease and racial/ethnic groups. Further research needs to examine the effect of adherence on OS and explore explicit benefits of metformin use in different racial and ethnic groups.

## Supporting information

S1 File Seer-Medicare cancer cohort selection. (DOCX)
