# Supplementary material for: Examining the relationship between metformin dose and cancer survival: A SEER-Medicare analysis
Source: PLoS One. 2022 Oct 19;17(10):e0275681. doi: 10.1371/journal.pone.0275681 (PMC9581409; doi:10.1371/journal.pone.0275681)
Supplement: S1 File — (DOCX) [file pone.0275681.s001.docx]

| **Step** | **Criteria** | **Inclusion** |
| --- | --- | --- |
| **1** | Diagnosis of lung, breast, colorectal, prostate, pancreas cancer from 2007 to 2016 | 1,269,374 |
| **2** | Only include clinical AJCC stage IV | 225,056 |
| **3** | Exclude if diagnosis was from death certificate or autopsy, or no pathological confirmation | 200,153 |
| **4** | Exclude if second cancer diagnosed within 60 months (Multiple cancer issue) | 191,362 |
| **5** | Only include Medicare Part A&B continuous coverage and exclude HMO coverage | 97,311 |
| **6** | Exclude if died within 90 days of the incident cancer diagnosis | 57,734 |
| **7** | Age at diagnosis: 66+ | 51,509 |
| **8** | Only include patients who were diagnosed with T2D and cancer at the same time (date of cancer diagnosis -30=< date of T2D diagnosis=< date of cancer diagnosis) | 7768 |
| **9** | Exclude developed T2D after cancer diagnosis | 7744 |
| **10** | Exclude metformin>=3000 mg/day | 7725 |

**Seer-Medicare Cancer Cohort Selection**
